# Myeloid Sarcoma: The Clinician's Point of View

**DOI:** 10.4061/2011/410291

**Published:** 2011-04-27

**Authors:** M. Malagola, M. Tiribelli, D. Russo, A. Candoni, G. Visani, A. Isidori

**Affiliations:** ^1^Department of Hematology, University of Brescia, P.le Spedali Civili 1, 25123 Brescia, Italy; ^2^Division of Hematology and Bone Marrow Transplant, Azienda University Hospital, Santa Maria della Misericordia Square, 33100 Udine, Italy; ^3^Hematology and Stem Cell Transplant Center, Marche Nord Hospital, Via Lombroso 1, 61122 Pesaro, Italy

## Abstract

Myeloid Sarcoma may occur in patients with an acute or chronic myeloproliferative disorder as well as de novo, with no apparent sign or symptom of concomitant haematological disease. The patients are preferentially young male and the site of disease localization may vary from central nervous system to pleura and thorax, with a common involvement of the reticuloendothelial system. The disease often shows chromosomal rearrangements, involving chromosomes 7, 8 and 3 and sometimes a complex karyotype (more than 3 abnormalities) is detected at diagnosis. The prognosis of this disease is dismal and only high-dose chemotherapy with autologous or allogeneic stem cells transplantation (auto or allo-SCT) may be potentially curative. In the absence of definitive elements that can define the prognosis of extra-medullary localization of “standard risk” AML, Clinicians should pursue the collection of data from different Centres and design of homogeneous treatment strategies, that could integrate standard chemotherapy with specific approaches, such as radiotherapy, transplant procedures or, in selected cases (such as those displaying molecular abnormalities involving protein tyrosine-kinases), molecularly targeted therapies.

 Recently Al-Khateeb et al. reported a clinicopathologic, cytogenetic, and outcome analysis of 21 adult patients with Myeloid Sarcoma (MS) [1]. Briefly, they show that MS may occur in patients with an acute or chronic myeloproliferative disorder (13 patients) as well as *de novo* (8 cases), with no apparent sign or symptom of concomitant haematological disease. The patients are preferentially young male, and the site of disease localization may vary from central nervous system to pleura and thorax, with a common involvement of the reticuloendothelial system. The disease often shows chromosomal rearrangements, involving chromosomes 7, 8, and 3, and sometimes a complex karyotype (more than 3 abnormalities) is detected at diagnosis. The authors confirm that the prognosis of this disease is dismal and that only high-dose chemotherapy with autologous or allogeneic stem cells transplantation (auto- or allo-SCT) may be potentially curative. 

From a clinical point of view, we agree with the authors' conclusions regarding the disease features and prognosis. As has been recently reviewed by Pileri et al. on 92 adult patients [2], development of a myeloid tumor at an extramedullary site can be either the sole evidence of a myeloid neoplasm or can happen concurrently or after an acute myeloid leukemia (AML) or other myeloproliferative neoplasms (MPN). In the former case (*de novo* MS), disease seems to be sensitive to radiotherapy and chemotherapy, while in the latter case (MS with concomitant AML/MPN) the outcome appears poor. Nonetheless, because of its relative rarity, AML with extramedullary localization poses a challenge to the clinicians, in particular for the definition of disease risk and for the choice of postinduction consolidation strategy (auto- or allo-SCT). These questions are more stringent when other clinical and biological features classify the AML in the good (e.g., normal leukocyte count, t(8;21), FLT3-ITD negativity and NPM positivity) or standard-risk group (e.g., normal leukocyte count, normal karyotype, FLT3-ITD negativity), as extramedullary localization could be regarded as the only high-risk feature of the disease [3, 4]. In this case, we think that an induction treatment with cytarabine, one anthracycline with or without a third drug (fludarabine or etoposide) and one or two consolidation treatment with high-dose cytarabine, could be the standard of care. In their manuscript, Al-Khateeb et al. report the remarkable rate of 70% complete remission (CR) with a “classical 3 + 7 regimen.” The problem is the intensification program: should the patient be addressed to auto-SCT or should a matched donor be identified and an allo-SCT performed? And, in this case, if a sibling donor is not available, should an alternative donor (e.g., matched unrelated donor (MUD) or partially matched cord blood) be searched and allo-SCT performed? Considering the poor long-term survival reported in the literature [2], we think that a young age (less than 55 years), good clinical conditions (no comorbidity), and availability of a well-matched sibling or MUD donor should suggest that an allo-SCT is performed when the patient is in first CR. In all other cases, an auto-SCT should be considered. An alternative approach could be aimed to define AML risk by testing as many prognostic factors as possible. In the last years, many new molecular markers have been shown to affect AML prognosis (e.g., CEBP-alpha mutations, MLL rearrangements, WT-1 expression, BAALC gene overexpression, and IDH2 mutations) [5]. The combination of different biological factors to define AML prognosis has been evaluated by Santamaría et al. who recently conducted a multivariate analysis on 9 molecular markers (ERG, EVI1, MLL-PTD, MN1, PRAME, RHAMM, WT-1, NPM, and FLT3) in 121 patients with cytogenetically normal AML (CN-AML) [6]. They proposed a biological scoring system that included EVI-1, PRAME, and ERG and that allowed patient stratification into four significantly different prognostic groups, both in the whole CN-AML population and in those patients with a typical intermediate prognosis (the FLT3-ITD negative/NPM negative and the FLT3-ITD positive/NPM positive) [6]. However, a possible limitation to this approach is that few centres are able to routinely perform analysis of three, four, or more biological markers. Moreover, the genetic assessment of different markers and interpretation of results are still not standardised, and this could cause some problems of data analysis and risk assessment.

In the absence of definitive elements that can define the prognosis of extramedullary localization of “standard risk” AML, we think that clinicians should pursue the collection of data from different centres and design of homogeneous treatment strategies, which could integrate standard chemotherapy with specific approaches, such as radiotherapy, transplant procedures, or, in selected cases (such as those displaying molecular abnormalities involving protein tyrosine-kinases), molecularly targeted therapies. 
